# Circadian Rhythm of Cardiovascular Disease: The Potential of Chronotherapy With Aspirin

**DOI:** 10.3389/fcvm.2019.00084

**Published:** 2019-06-20

**Authors:** Marleen Buurma, Jeske J. K. van Diemen, Abel Thijs, Mattijs E. Numans, Tobias N. Bonten

**Affiliations:** ^1^Department of Public Health and Primary Care, Leiden University Medical Center, Leiden, Netherlands; ^2^Department of Internal Medicine, Amsterdam University Medical Center, Amsterdam, Netherlands

**Keywords:** aspirin, cardiovascular disease, platelet aggregation, circadian rhythm, chronotherapy

## Abstract

Almost all the systems in our body adhere to a daily 24 h rhythm. The cardiovascular system is also affected by this 24 h rhythm. In the morning there is a change in various cardiovascular processes, including platelet aggregability. These changes may play a role in the relative excess of early morning cardiovascular events. The number of recurrent cardiovascular diseases (CVD) could, in theory, be reduced by responding to this 24 h rhythm with timed medication intake (chronotherapy), which also applies to aspirin. Multiple studies on chronotherapy with low-dose aspirin are promising, showing a decrease in early morning platelet activity with evening intake compared with morning intake. However, in order to further demonstrate its clinical impact, randomized trials with cardiovascular events as a primary outcome are needed. This review discusses the available evidence of the effects of circadian rhythm on CVD and the potential positive effect of chronotherapy with aspirin.

## Introduction

In the past centuries, more and more has been discovered about the different mechanisms in our body as well as how to apply this in our medical treatment regimes. This article will discuss one of those discoveries, namely the circadian rhythm and its influence on the development of acute events of cardiovascular disease (CVD). Platelet aggregation is one of the mechanisms responsible for the development of adverse cardiovascular events. In current medicine, antiplatelet medication is the cornerstone of prevention of recurrent cardiovascular events. Recent studies assessed whether optimal timing of intake of medication, such as aspirin, can give a further reduction of CVD.

## The Discovery of Aspirin and Circadian Rhythms

Around 2,400 years ago Hippocrates was one of the first who described the analgesic effects of willow bark (1). Aspirin, a medicine derived from the willow bark, is still one of the most widespread used medications in the world (2). It is sometimes called the wonder drug, because of its different properties like antipyretic, analgesic, antiplatelet, anti-inflammatory, and even anti-cancer effects (1, 3).

These observations were already made well before the use of evidence-based medicine. In 1563, Reverend Edward Stone carried out a carefully planned clinical study on ~50 patients suffering from inflammatory disorders and pains with encouraging results (1, 4). This research provided the first scientific basis for the common use of willow bark.

The next step in the scientific discovery was the isolation of the active ingredient of the willow bark (1). In 1897, acetylsalicylic acid was created by Felix Hoffman, by modifying salicylic acid by acetylation, and named it Aspirin. This acetylated salicylic acid also provided for the properties of Aspirin to prevent (recurring) CVD events (4).

In 1950, before the exact working mechanism of aspirin was known, the family doctor Lawrence Craven wrote about his experience with aspirin in a number of American journals. In one of his articles, he mentioned that in patients using a higher dosage of aspirin impregnated chewing gum than that he prescribed after undergoing a tonsillectomy, more complications such as bleeding occurred. Following this, he reasoned that aspirin might have a thrombolytic effect (1).

Twenty years later (1971), Vane and co-workers discovered that aspirin inhibits the synthesis of prostaglandin. This mechanism explains the antipyretic, anti-inflammatory, and antiplatelet effects of aspirin (5). For this discovery, Vane received the Nobel prize for Medicine in 1982. Even today, scientists are discovering new properties of aspirin and are reassessing its value in the light of new knowledge of mechanisms in pathophysiology. This includes recent knowledge about circadian rhythms in our body, which seems a new opportunity to optimize today's medical treatment for CVD.

For a long time, it was thought that almost everything in our body occurred randomly. In 1984, a gene was identified in fruit flies, that ensures the 24 h rhythm (circadian rhythm) in physiological processes, like hormone concentrations (6). The circadian rhythm in physiology and human behavior (e.g., sleep, activity, and eating), are essential for all organisms enabling them to anticipate and adapt to the natural environment (7, 8).

This circadian rhythm is controlled by the central or master clock and peripheral clocks. The central clock situated in the hypothalamus in the central nervous system, called the suprachiasmatic nucleus (SCN), coordinates the expression of the clock genes throughout the body (9). This central clock is mainly driven by the alternation of light and dark (10, 11). The peripheral clocks can be found in almost any tissue of the body, including the cardiovascular system (11, 12). An example of one of those genes is the CLOCK gene. This gene affects, among other things, platelet aggregation, and the expression of plasminogen activator inhibitor-1 (PAI-1). A mutation in the CLOCK gene can result in several changes. For example, the daily variation in platelet aggregation disappeared in CLOCK mutant mice. Besides, mice with CLOCK mutation had reduced and non-rhythmic secretion of PAI-1 by the endothelial cells (7).

The internal clock has a lot of influence on the functioning of our body. Chronic disruption of this clock, for example by shift work, is thought to be related to metabolic syndrome, cancer, diabetes, and cardiovascular disease (7, 9, 13).

## Cardiovascular Disease and Circadian Rhythm

The human cardiovascular system has different activity patterns with cycles of 24 h, including heart rate, blood pressure, circulating catecholamines, blood coagulation markers, vascular endothelial function, and autonomic nervous system (8, 14). In the morning there is a change in a number of cardiovascular processes (Table 1). Importantly, these rhythm modifications in the morning correspond to the occurrence of CVD. An apparent increase in the number of adverse cardiovascular events is observed during morning hours (06.00–12.00 h), such as stroke, myocardial infarction, ventricular arrhythmias, and sudden cardiac arrest (8, 21, 22). Patients who had a heart attack during morning hours will mostly have a larger infarct size and worse prognosis, compared with patients having a myocardial infarction during the rest of the day (23).

**Table 1:** Important changes in cardiovascular processes during the morning.

**Cardiovascular processes**	**Activation during morning hours**	**Negative effect on cardiovascular disease**
Sympathetic tone	Increased	Increase in blood pressure, heart rate, and vasoconstriction (15).
Parasympathetic function	Decreased	Increase in heart rate and vasoconstriction (15).
Blood pressure	Increased	There is an associations between an increase in blood pressure and ischemic heart disease and stroke (16).
Heart rate	Increased	High resting heart rate is associated with adverse cardiovascular events (17).
Vascular endothelial function	Decreased	Endothelial dysfunction is associated with atherosclerotic plaque formation (18).
Platelet aggregation	Increased	A higher platelet aggregation increases the change of the formation of blood clots and thereby, adverse cardiovascular events (19).
Thrombolytic activity	Decreased	Lower thrombolytic activity increases the change of the formation of blood clots, and thereby adverse cardiovascular events (20).

## Autonomic Nervous System and Circadian Rhythm

The autonomic nervous system plays a vital role in physiological and pathological responses of the cardiovascular system. The sympathetic system induces an increase in heart rate, myocardial contractility, and peripheral resistance. The parasympathetic system, on the other hand, has the opposite effect, where it primarily causes a reduction in heart rate and, to a lesser extent, a decrease in cardiac contractility (15). The parasympathetic and sympathetic system both follow a circadian rhythm. Whereas, the parasympathetic activity is more pronounced during the night, the sympathetic system has a peak in activity during the morning. Moreover, this early morning peak coincides with a high sensitivity of vascular receptors (14, 21). This morning peak of the sympathetic nervous system and the increased activity of the renin-angiotensin-aldosterone axis along with the decreased parasympathetic system contribute to the rise in blood pressure and heart rate in the morning (21). These changes in the morning probably add to the increased incidence of CVD during the same time period (8).

## Blood Pressure and Circadian Rhythm

Blood pressure follows a 24 h rhythm. In the morning there is a rise, which reaches a plateau around 11 am. Then it gradually decreases, and around midnight it reaches its lowest value (24). Blood pressure usually is about 10 to 20% lower while sleeping than during daytime (25). The change in blood pressure is related to the change in activity of the sympathetic nervous system running parallel to the normal sleep-wake cycle (24). Night-time blood pressure is lower due to the reduction of sympathetic tone and the parallel increase in vagal activity during the sleep period. External factors influence blood pressure as well, such as physical activity, emotions, intake of food and drinks, and the variation in light and dark (20).

The time frame of the rise in blood pressure in the morning coincides with the increase in the number of cardiovascular events in the morning (8). Due to the rise in blood pressure in the morning, vulnerable plaques in arteries are more prone to rupture, which can lead to acute adverse cardiovascular events (26). Not only the morning blood pressure rise increases the risk of developing CVD. Research has shown that having continues high blood pressure during nighttime (non-dipping) increases this risk as well. As a matter of fact, it is the lack of a nightly drop in blood pressure which is a risk of the development of CVD (8, 24). The MAPEC study showed that, with the use of chronotherapy, nighttime blood pressure would be reduced, thereby diminishing the risk of CVD (27).

## Vascular Endothelium and Circadian Rhythm

The vascular endothelium has anti-atherosclerotic functions, plays a role in the aggregation of platelets and regulates the patency of vessels via excretion of nitrogen oxide (8, 28). All of these factors influence the cardiovascular system, and therefore, dysfunction may lead to adverse cardiovascular events (8). Dysfunction of the endothelium results in an imbalance in the production and consumption of nitrogen oxide (18). The endothelium-dependent vasodilator function is normally reduced during morning hours, because of the lower production of nitrogen oxide (20). This vasodilating function of the endothelium protects against diseases such as hypertension, stroke, and myocardial infarction. The deterioration of this function might contribute to the morning peak of CVD (8, 28, 29).

## Blood Clotting and Circadian Rhythm

The ability of blood to form clots can be life-saving during a bleeding event. On the other side, the formation of clots in blood vessels can also lead to ischemic stroke, myocardial infarction, and sudden cardiac arrest. As a counterpart to the development of blood clot, there is thrombolysis, which helps to break down clots and keep blood vessels open. The balance between clotting and bleeding tendency changes during the day. Coagulation is increased during morning hours, partly because platelet aggregation and platelet surface activation markers experience a peak in the morning between 06.00 and 12.00 h (2, 30, 31). In this same period, there is reduced thrombolysis which can partly be explained by a reduced concentration of plasmin-plasmin inhibitor complex in blood during morning hours (20). Secondly by an increased level of plasminogen activator inhibitor-1 (PAI-1). PAI-1 is one of the most important inhibitors of plasma fibrinolytic activity. So, increased PAI-1 concentrations enhance the chance of the development of blood clots (32, 33). These phenomena lead to an increased risk of developing blood clots in the morning hours, possibly contributing to the peak of CVD in this period (2, 8, 33).

The development of CVD is influenced by factors such as continuous interaction between the various circadian phases in the body, the behavior of the individual such as physical exercise and personal risk factors. The circadian rhythms of different processes in the body, such as vagal withdrawal, sympathetic activation, and increased blood clotting, have a homeostatic advantage in most circumstances, anticipating the expected behavioral changes in the morning, and other periods of the day. But in a person susceptible to CVD, the circadian change in the morning can exceed the theoretical risk threshold, which leads to the development of adverse cardiovascular events. The question is whether the circadian rhythm is actually the cause of CVD or whether it reveals the underlying vulnerability (8, 33).

Based on currently available evidence we believe that the circadian rhythms and their influence on CVD, as described above, should be taken into account in the treatment of CVD. Also, the pharmacokinetics of the drug is maybe influenced by this rhythm (34). By reckoning the importance of the circadian rhythm in total during treatment, this might lead to increased effectiveness and fewer side effects. Furthermore, the effectiveness of medicines can also be influenced by comedication. A recent study shows that coadministration of low-dose aspirin and atorvastatin, and possibly other statins, reduces cardiovascular events (35). Still, an important role may be reserved for using the circadian rhythm for drug treatment, such as with acetylsalicylic acid.

## Aspirin and Time of Intake

Platelets are key players in the development of arterial thrombosis. Consequently, aspirin is a cornerstone of secondary prevention because it inhibits platelets. The majority of patients take maintenance doses of 75–325 mg once a day in the morning, after awakening (36). However, research shows that, despite the improvement of lifestyle and drug prevention, 10–33% of these patients have a recurrence of an adverse cardiovascular event within 5 years after their first event (37). The reasons for this recurrence is not completely clear, but probably multifactorial. In theory, it could be partially explained due to the pharmacokinetic properties of aspirin and the circadian physiology of platelets. Aspirin is absorbed reasonably quickly with a Tmax of 0.5–2 h for non-enteric-coated aspirin and 3–4 h for enteric-coated aspirin. But also rapidly excreted with a half-life of acetylsalicylic acid of ~20 min (38). The current once-daily regimen is associated with a slow increase in platelet activity within the 24 h dosing interval (39–41). A likely explanation for this phenomenon could be the production of new platelets (42). These new platelets are released at a rate of 10–15% per day (43, 44). Because of the pharmacokinetic properties of aspirin, the cyclooxygenase-1 (COX-1) of the newly released platelets is not inhibited, and so they are able to form blood clots. Previous studies have shown that 95% of all platelets' COX-1 must be inhibited to achieve effective reduction of platelet aggregation (45, 46). This percentage is supported by a recent study in which insufficient inhibition of platelet aggregation was seen 24 h after morning aspirin intake in 25% of patients with CVD (40). The production and delivery of these new platelets follow a circadian rhythm with peak release in the late night and early morning (47). This, too, coincides with the occurrence of adverse cardiovascular events (8, 21, 22). Therefore, it can be essential to ensure adequate platelet inhibition during the morning hours. By taking the aspirin on awakening, 10% uninhibited platelets are present during the morning hours. By responding to the circadian rhythm and taking aspirin at bedtime, the proportion of unrestrained platelets during the morning hours would theoretically be reduced to 5% (2). This results in increased inhibition of the platelet reactivity during morning hours, and possibly a reduction in adverse cardiovascular events.

Several studies try to accomplish a further decrease in the number of recurrences of adverse cardiovascular events by further reducing the number of uninhibited platelets. These studies are assessing the effect on platelet aggregation by increasing dosage, increasing intake frequency, and the use of chronotherapy.

### Increasing Aspirin Dosage

When comparing standard morning low-dose aspirin with a higher dosage on platelet aggregation, the effectiveness of the increase differ. In most studies, the inhibition of the sTxB2 shows sufficient inhibition at a dosage between 80 and 325 mg aspirin per day (48–52). However, three trials reported higher sTxB2 inhibition after dosages above 325 mg of aspirin in comparison with a standard once daily low-dose regimen (52–54). There was a small to no increase in platelet inhibition measured by the Light Transmission Aggregometry if the dosages were above 325 mg (52, 54–57). The results of platelet aggregation measured with the Platelet Function Analyzer and VerifyNow were in line with the sTxB_2_ results: two trials demonstrated sufficient levels of platelet inhibition with once-daily dosages of 75–325 mg of aspirin (50, 56, 58).

### Increased Intake Frequency of Aspirin

Regarding the increase of the intake frequency, a higher sTxB2 inhibition was reported in three studies, when intake was increased to more than one intake per day in comparison with the standard once daily low-dose regimen (50, 59, 60). Capodanno et al. reported a superior inhibition when intake was increased to more than once-daily of aspirin in comparison with the standard once-daily dosing, measured by the Light Transmission Aggregometry and VerifyNow (50). On the other hand, five trials did not demonstrate superiority in the level of inhibition by increasing the intake frequency of aspirin (49, 59, 61–63). In addition, when measuring the platelets aggregation by Platelet Function Analyzer, a difference was seen between patients with stable CVD and healthy volunteers (59, 64). In patients with stable CVD, a significant decrease in platelet aggregation was seen when comparing intake of once daily with twice a day (64). This difference was not demonstrated in healthy volunteers (59).

### Chronotherapy of Aspirin

Two randomized cross-over trials on chronotherapy concerning platelet function inhibition suggested a superior sTxB_2_ inhibition after once-daily evening intake of low-dose aspirin in comparison with morning intake (2, 59). van Diemen et al. reported an increased platelet inhibition, measured with Light Transmission Aggregometry, after low-dose aspirin intake in the evening in comparison with the standard low-dose aspirin once daily in the morning (65). When measuring the platelets aggregation by Platelet Function Analyzer, a different effect on platelet aggregation was found between patients with stable CVD and healthy volunteers. In stable cardiovascular patients, a statistically significantly higher level of platelet inhibition was demonstrated after evening intake, whereas a trial conducted in healthy subjects did not show a difference (59, 65).

One parallel RCT and three randomized cross-over trials reported more platelet inhibition, measured by the VerifyNow, after evening intake of low-dose aspirin compared with the standard once daily morning dosing (2, 59, 66, 67).

### The Different Intake Regimens

When analyzing these different studies on platelet aggregation, one could conclude that an aspirin dosage between 75 and 325 mg once daily provides sufficient platelet inhibition in early morning hours (48–52, 56, 58, 59, 61–63). Especially when we consider that changes in the dosage, frequency, or intake time, in addition to their positive effect on platelet aggregation, may also give a higher chance of side effects. When increasing the dosage above 325 mg once daily minor improvement of platelet inhibition is obtained (52–56). Besides, the risk of side effects such as gastrointestinal bleeding increases with increasing dosage (68). Increasing the intake frequency seems also beneficial on the level of platelet inhibition (50, 59, 60, 64). Again, the total daily dose should not exceed 325 mg, due to the increased chance of complications. Beside, adherence deteriorated with an increasing number of intake moments (69). Importantly, the application of chronotherapy showed, in most of the studies, a higher level of platelet inhibition when comparing the evening intake with the standard once-daily morning intake (2, 59, 65–67). The chance of side effects with the use of low-dose aspirin is similar between the different intake regimens (70). But there have been few long-term studies on the effects of increased intake frequency and chronotherapy so far. However, the evidence for chronotherapy seems hitherto to be the most consistent compared with the other interventions (increasing dosages and intake frequency). Chronotherapy probably has a lower expectancy of side effects on the long-term, because there is no increase in dosage.

## Conclusion

It is clear that the cardiovascular system of our body follows a circadian rhythm. Several studies have shown that adverse cardiac events also follow a circadian rhythm, with a vulnerable period in the early-morning hours. The described circadian processes of the cardiovascular system, each with its own rhythm, presumably all contribute to the development of adverse cardiac events. It is essential to investigate how the medical profession can respond to this circadian rhythm in the treatment of CVD leading to increased effectiveness of treatments and/or fewer side effects. The available evidence already shows positive effects of chronotherapy, for instance by reducing nighttime blood pressure. Also, recent studies on chronotherapy suggest that low-dose aspirin taken at bedtime compared with intake on awakening can improve platelet inhibition during morning hours. There is a need for randomized controlled trials with cardiovascular events and side-effects as endpoints, to further supports the promising chronotherapy hypothesis.

## Author Contributions

MB, TB, JD, AT, and MN made substantial contributions to conception and design and gave final approval of the version to be submitted and any revised version. MB, JD, and TB participated in drafting the article. TB, AT, and MN participated in revising it critically for important intellectual content.

### Conflict of Interest Statement

The authors declare that the research was conducted in the absence of any commercial or financial relationships that could be construed as a potential conflict of interest.
